# Age-Related Changes in the Behaviour of Domestic Horses as Reported by Owners

**DOI:** 10.3390/ani10122321

**Published:** 2020-12-07

**Authors:** Bibiana Burattini, Kate Fenner, Ashley Anzulewicz, Nicole Romness, Jessica McKenzie, Bethany Wilson, Paul McGreevy

**Affiliations:** 1 Sydney School of Veterinary Science, Faculty of Science, University of Sydney, Camperdown, NSW 2006, Australia; kate@kandooequine.com.au (K.F.); aanz4186@uni.sydney.edu.au (A.A.); nrom3994@uni.sydney.edu.au (N.R.); Bethany.wilson@sydney.edu.au (B.W.); paul.mcgreevy@sydney.edu.au (P.M.); 2 School of Life and Environmental Sciences, Faculty of Science, University of Sydney, Camperdown, NSW 2006, Australia; jmck2527@uni.sydney.edu.au

**Keywords:** temperament, trainability, boldness, independence, welfare, rider safety

## Abstract

**Simple Summary:**

Some treatments for common problem behaviours in domestic horses can compromise horse welfare. Such behaviours can be the manifestation of pain, confusion and conflict. In contrast, among the desirable attributes in horses, boldness and independence are two important behavioural traits that affect the fearfulness, assertiveness and sociability of horses when interacting with their environment, objects, conspecifics and humans. Shy and socially dependent horses are generally more difficult to manage and train than their bold and independent counterparts. Previous studies have shown how certain basic temperament traits predict the behavioural output of horses, but few have investigated how the age of the horse and the age it was when started being trained under saddle affect behaviour. Using 1940 responses to the Equine Behaviour Assessment and Research Questionnaire (E-BARQ), the current study explored the behavioural evidence of boldness and independence in horses and how these related to the age of the horse. Results revealed age-related effects on boldness and independence of horses. Older horses were bolder than younger horses, but horses started under saddle at an older age were less bold and independent than those started at a younger age. Additionally, significant differences in boldness and independence relating to specific breeds and primary equestrian disciplines also emerged. Finally, riders with eight or more years of riding experience reported having more independent horses than those who had ridden their whole lives. Understanding how horses’ ages affect behavioural traits can improve horse–rider matching and potentially also optimise welfare.

**Abstract:**

The broad traits of boldness and independence in domestic horses can affect their usefulness and, indirectly, their welfare. The objective of the current study was to explore associations between attributes that reflect equine boldness and independence with both the age of horses and the age at which they were started under saddle, as well as other variables including breed, colour and primary equestrian discipline. All data were sourced from responses (*n* = 1940) to the 97-question online Equine Behaviour Assessment and Research Questionnaire (E-BARQ). Twenty E-BARQ items from the dataset were selected to reflect boldness and independence and were tested for univariate significance at *p* < 0.2. Multivariable modelling of the effect of age on remaining traits was assessed by an ordinal logistic regression, using a cumulative log odds model. This revealed that older horses were bolder (*p* = 0.012). However, horses started under saddle at an older age were less bold and less independent (*p* = 0.040 and *p* = 0.010, respectively). Australian Stock Horses were bolder and more independent (*p* = 0.014 and *p* = 0.007, respectively) than crossbreed horses. Horses used for breeding conformation (*p* = 0.039), working equitation (*p* = 0.045), eventing (*p* = 0.044) and traditional working horses (*p* = 0.034) were bolder than those used for other disciplines. Dressage (*p* = 0.039) and therapy (*p* = 0.040) horses were less bold than horses used for other disciplines. Stallions were bolder (*p* = −0.034) than geldings. Brown (*p* = 0.049) and chestnut (*p* = 0.027) horses were less bold than bay horses. Compared to crossbreed horses, Thoroughbreds (*p* = 0.000) and companion horses (*p* = 0.017) were less bold whilst heavy horses (*p* = 0.029) and ponies (*p* = 0.044) were bolder. Compared to pleasure horses, mounted games horses (*p* = 0.033) were less independent whereas working equitation horses (*p* = 0.020) were more independent. Riders with more than eight years’ experience reported more independence in their horses (*p* = 0.015) than those who had ridden their whole lives. The study findings suggest that boldness and independence are separate traits and only boldness was associated with the age of the horse. Factors that relate to desirable boldness and independence are important in ridden horses because they can affect rider safety. Results from this study should improve horse–rider matching and thereby potentially enhance horse welfare.

## 1. Introduction

Horses, similar to many other domestic species, have occupied many roles in the human domain. Historically, in wild and early managed contexts, they were used primarily as a food source. However, their domestication has seen them deployed in transport, agriculture and military contexts [1], serving in roles they have occupied for centuries. Today, horses are found in various additional contexts, ranging from use in competition (e.g., racing and other performance sports), leisure riding and providing companionship [2].

The ability of an individual domestic horse to navigate its environment and respond to novel stimuli with confidence influences its economic value to trainers and owners [3]. Thus, horses with low confidence that impairs trainability or renders them problematic to handle may be of less commercial value than those that are easier to handle and train [4,5,6]. Horses that are problematic to handle, train and ride are often sold repeatedly. In the process, they can be subjected to increasingly harsher training methods as they change hands [7]; methods that can exacerbate the undesirable behaviours and commonly increase stress [7]. Thus, the welfare of these horses may be compromised [7,8,9,10]. Indeed, many such horses are culled as a result of their behaviour [7].

When animals are being trained by humans, their temperament always has a strong influence on outcomes [11]. Equine temperament (also sometimes called personality or temperament) can have a major influence on the welfare of horses in training and horses’ ability to adapt to management regimes [12,13]. It has been shown that temperament is a top criterion used by riders, breeders and trainers when assessing the breeding, trainability and rideability of horses for all disciplines [14]. Unsurprisingly, it is also critical when making purchasing and marketing decisions [14].

In any managed species, operant conditioning outcomes can be influenced by the shy–boldness continuum [15,16,17,18,19,20,21]. For example, in working dog trials, performance correlates with boldness [11]. Boldness is consistent with an active and assertive personality, sometimes labelled extraversion [22]. In horses, it is described as a general lack of fear and is a highly valued trait [23]. Boldness is also the propensity of an animal to take risks, such that a high likelihood of risk-taking is regarded as a sign of boldness, while a low likelihood is regarded as a sign of shyness [24]. Boldness helps ridden or driven horses to pass obstacles they encounter in their work without a loss of line, i.e., without deviating from the trajectory determined by the rider or driver [25]. Clearly, this can relate directly to horses’ performance in ridden contexts. For example, in show-jumping, horses must show sufficient boldness to navigate over forbidding jumps that other horses would circumvent or baulk at [23]. Bold horses are more adaptable and are better able to cope in new environments as they are more exploratory and less anxious than their shyer counterparts [26].

Among social species, shy individuals often remain with conspecifics when foraging to benefit from shared surveillance of the immediate environment for threats and thus a reduced risk of predation. In contrast, studies of sheep have shown that bold individuals may forage at a distance from conspecifics to take advantage of reduced grazing competition [27]. Thus, it is not surprising that those animals that most often initiate such departures from the group tend to be relatively bold [26].

A separate trait, independence, is defined as being self-controlled and not excessively bonded to other horses or humans [28]. It encompasses self-reliance which reflects the ability to be at ease when left alone, away from the herd [29,30]. Horses are gregarious animals by nature, and social isolation from conspecifics can trigger overt behavioural responses, such as vocalisation [31,32]. Gregariousness can be considered similar to dependence, and the opposite of independence. Lansade et al. [31] described gregariousness as reactivity to social isolation from conspecifics. Suwala et al. [33] used the term independence interchangeably with self-reliance. Horses that are comfortable in social isolation may be those that are most relaxed, self-reliant and independent [34].

Behavioural changes in horses are known to accompany increasing maturation and reflect acquired experience and accumulated familiarity with different situations [1,8]. At its simplest level, relevant familiarisation begins in neonates with introductions to novel stimuli and is enhanced by foals’ predisposition to play [8]. In horses, such experiences help shape social and performance skills [35,36]. For example, it has been demonstrated that foals under one year of age exhibit fewer fear responses and more interest in novel stimuli (notably when exposed to a regular feed-box and a feed-box covered with a black cloth) than horses aged between 2 and 15 years [37]. This suggests that, for each task, there may be an optimal time for learning and, by inference, there may be a preferred window period for early handling and foundation training in horses [3].

Experiences during particular developmental stages can influence a horse’s behaviour later in life. For example, age at weaning and quality of maternal care may influence the development of stereotypic behaviours in horses [38]. Hand-reared orphan foals are less emotional when placed in a novel environment than naturally reared foals. These same foals often show virtually no fear of humans and may be difficult to handle as adult horses [35,39]. Henry et al. [40] reported that simply allowing foals to observe their dams being handled agreeably can have a calming effect on them in later life. For example, such handling of the dams in the first two days after parturition, improved foal–human relationships in the long-term [41]. Yearling horses that had been handled as sucklings learned faster than both horses that had almost no handling (except for haltering for three days as foals and weaned at 8 months) and those that were extensively handled (housed in pens, haltered at three months and handled at least four times per week) [42]. This suggests that handling young horses at a particular time during their development can optimise reactivity later in life [40].

Understanding the relationships among age, temperament and undesirable behaviours is important for the training, housing and breeding of horses [12]. Sackman et al. [12] reported that, compared to horses aged between 6 and 10 years (*n* = 264) and those older than 20 years (*n* = 101), horses aged between 11 and 15 years (*n* = 229) were ranked by survey respondents as the most nervous in any situation they encountered. This is may be due to the fact that horses aged 20 years and older have encountered serial changes in ownership, environment, rider and increased time being transported and competing. Younger horses are generally yet to be exposed to enough novel stimuli to generalise their habituation and moderate their innate fear and flight responses, whilst the older horses are regularly habituated and desensitised to multiple stressors and novelties. The lack of linearity in the relationship between nervousness and age in the Sackman et al. study confirms the importance of evaluating longitudinal flux in interactions between temperament and optimal performance [12].

Beyond age, breed, training and experience are important influences on behaviour and so must be considered when evaluating equine temperament [43]. Even housing and feeding can affect the behaviour of horses [44], so it pays to gather data on current management factors when investigating a horse’s behaviour.

The goal of the current study was to explore how the traits that reflect boldness and independence in domestic horses change with age, using owner-reported observations. Data were gathered from horse owners and handlers around the world, via the ongoing Equine Behaviour Assessment and Research Questionnaire (E-BARQ) project [45]. The study aimed to address relevant knowledge gaps by exploring associations between attributes that reflect equine boldness and independence and the current age of the horses as well as the age at which ridden horses were started under saddle. In addition, the effects on these temperament traits of horse breed, age and colour as well as the respondents’ experience and preferred equestrian discipline were also examined.

## 2. Materials and Methods

### 2.1. Ethics Committee Approval

This project was created and piloted by the University of Sydney with approval from the University of Sydney Human Research Ethics Committee (approval number: 2012/565).

### 2.2. Questionnaire Design

The questionnaire consisted of 97 matrix-style questions within the E-BARQ [45]. These questions consisted of 42 demographic and behavioural items relating to both horses and their owners/handlers. These items were divided into ridden (horse had been ridden in the last 6 months) and non-ridden (horse had not been ridden in the last 6 months) questionnaires. A copy of the entire questionnaire appears in Supplementary File 1.

### 2.3. Data Collection

Data were collected using the E-BARQ questionnaire [45], a validated equine behaviour assessment instrument [46]. The questionnaire was built using Qualtrics XM survey software (Qualtrics Labs Inc, Provo, UT, USA) and accessed online by participants via a web link. Respondents created an account prior to undertaking the survey (https://www.e-barq.com/), enabling them to “save and return” to complete the survey later, if required. They were directed to report on behaviours the individual focal horse had exhibited in only the past six months.

After completion of the questionnaire, respondents were provided with a *Share-&-Compare* graph of their horses’ results [47]. The E-BARQ questionnaire was distributed via social media posts (including Facebook and Instagram), and through email lists of Pony Clubs Australia (https://ponyclubaustralia.com.au/), Horse and Peoples Magazine (https://horsesandpeople.com.au/), Equitation Science International (https://www.esi-education.com/) and Kandoo Equine (https://www.kandooequine.com/) to a wide potential range of horse enthusiasts. A total of 2001 responses were collected for the current study. Of those responses, 61 had provided no age for the horse, and so these were not included in the analysis. Therefore, a total of 1940 responses were used in the current study.

Respondents came from 33 different countries and reported on 78 different breeds of equids. Data were comprised of 58% geldings and 38% mares, with the remainder being stallions, colts and fillies. Respondents were mostly experienced riders, with 83% having been riding for more than eight years. Respondents were asked “*what is this horse’s main discipline*” and invited to choose from 42 riding and handling disciplines.

### 2.4. Trait Selection

#### 2.4.1. Determination of Number and Composition of Dependent Indices

A rotated principal component analysis was conducted on the original E-BARQ pilot [45]. This type of analysis classifies correlated variables, allowing for the discovery of independent latent, not directly measured, variables. Twenty E-BARQ items (exploring both ridden and unridden responses are combined) were analysed in five underlying temperament (T) components of interest: T1 (Does the horse strongly avoid, shy or bolt away from bicycles, cars, chainsaws, horse and carts, motorbikes or umbrellas?); T2 (When left alone or taken away from other horses, does the horse box-walk, canter when loose, fence-walk, paw, sweat, tremble or vocalize?); T11 (When left alone or taken away from other horses, does the horse pull back when tied?); T18 (Does the horse strongly avoid plastic bags or get distracted by unfamiliar sights?); T20 (Does the horse strongly avoid, shy or bolt away from plastic bags or dogs?). To combine these into fewer relatively uncorrelated indices, a parallel analysis, comparing the scree of factors of the standardised observed data with that of a random data matrix of the same size, was carried out using the Psych package, version 1.9.11 (Procedures for Personality and Psychological Research, Northwestern University, Evanston, IL, USA) of R statistical software, version 4.0.0 (R Foundation for Statistical Computing, Vienna, Austria).

#### 2.4.2. Construction of Dependent Indices

Boldness and independence indexes were constructed by assigning a numerical value to scores on a five-point Likert scale for the relevant E-BARQ items and summing these values together. In the case of missing values, the sum was divided by the number of E-BARQ items in the index for which information for that horse was available and multiplied by the number of items used to calculate the index, weighting the missing value according to the horse’s score for similar items rather than imputing an overall mean. If no E-BARQ items for an index were completed, then a value for that horse was not calculated (using the statistical package: R statistical software, version 4.0.0, R Foundation for Statistical Computing, Vienna, Austria).

### 2.5. Independent Variables

For *age-of-handling* versus *age-started-under-saddle*, the main predictor variables of interest were *age-of-horse*, *age-when-handling-began* and *age-started-under-saddle*. A Fisher exact test was performed. For *age-of-handling* versus *age-of-horse*, an ANOVA wrapper from the car packages was performed. Predictors with *p* < 0.2 on univariate analysis were passed into the multivariate model building process.

### 2.6. Multivariate Modelling

#### 2.6.1. Model Building Boldness

The final model for boldness was:

Boldness ~ Age of the horse + Gender + Sex of the horse + the analysis of the colour of the horse + the analysis of the Breed of the horse + the age the horse was Started under saddle_/the analysis of the Discipline the horse was worked in

The Q-Q plot and scale location plot demonstrated skew and heteroscedacity of the residuals. Inversion of the index, followed by a square-root transformation, sufficiently corrected these concerns. The index was also then multiplied by −1, for ease of interpretation.

#### 2.6.2. Model Building Independence

The final model for independence was:

Independence ~ Age of the horse + Age of the rider + Sex of the horse + Analysis of the colour of the horse + Rider Experience + age horse was Started under saddle/the Analysis of the Breed of the horse + the age the horse was Started under saddle/ the Analysis of the Discipline the horse was worked in

The Q-Q plot and scale location plot demonstrated skew and heteroscedacity of the residuals. Inversion of the index, followed by a square-root transformation, sufficiently corrected these concerns. The index was also then multiplied by −1, for ease of interpretation.

## 3. Results

Responses were received from 2001 participants. Of these 2001, 61 had not provided age responses for the horse and were not included in the analysis. Therefore, only 1940 responses were analysed.

### 3.1. Trait Selection and Independent Variables

This parallel analysis suggested three underlying components. When three principal components were extracted, using the psych package and rotated using a varimax rotation, to facilitate interpretation, a series of loadings emerged. These can be seen in Table 1.

Because it was noted that the response rate for two of the T11 questions was substantially lower (34.78–34.83%) than for the other E-BARQ items (>90%), removal of these questions was considered, and the parallel analysis was repeated without them. This dropped the number of components suggested by the parallel analysis to two, the loadings for which are shown in Table 2.

Kaiser, Meyer, Olkin Measures of Sampling Adequacy (MSA) and Cronbach’s α were also considered. For T2 alone, the MSA value was 0.86 and Cronbach’s α was 0.85 (95% confidence interval (CI) = 0.83–0.86). When the relatively complete T11 of pulling back when alone was added to T2, both sampling adequacy (MSA = 0.88) and internal consistency (α = 0.86; 95% CI 0.85–0.87) improved. When the remaining T11 items were also added, the overall MSA was reduced slightly to 0.85, but internal consistency remained stable at 0.86 (95% CI 0.86–0.87). Based on these findings, the decision was taken to retain all items from factors T2 and T11 for the Independence personality trait.

T1, T18 and T20 items had an overall MSA of 0.89. The overall internal consistency was 0.88, and the analysis suggested no improvement in internal consistency following the dropping of any single item. Based on these findings, the decision was taken to retain all items from factors T1, T18 and T20, for the Boldness personality trait.

For *age-of-handling* versus *age-started-under-saddle*, a Fisher Exact test showed that these variables are not independent of each other (*p* < 0.0001). For *age-of-handling* versus *age-of-horse*, an ANOVA wrapper from the car packages showed that these variables are not independent (*p* = 0.0085). An ANOVA wrapper from the car package did not provide evidence that these variables are not independent and could be considered for the same model. Because of the collinearity of *Age-of-Handling* with both *Age-of-horse* and *Age-started-under-saddle*, the remainder of the analysis focused on the latter two variables. Other predictor variables available (including potential confounders) were assessed for potential inclusion in the final model by univariate analysis and appear in Table 3.

### 3.2. Boldness

Analysis of the coefficients revealed that the age of the horse was significantly associated with boldness, in that older horses were bolder than younger horses (*p* = 0.012). Horses started under saddle late were less bold than those started under saddle at an earlier age (*p* = 0.040). Furthermore, Australian Stock Horses were bolder than crossbred horses when started under saddle at an older age (*p* = 0.014). Horses used for breeding conformation, working equitation, eventing and working cow horses were bolder than pleasure horses (*p* = 0.039, *p* = 0.045, *p* = 0.044 and *p* = 0.034, respectively) when started under saddle when older. Horses used for dressage and therapy horses were less bold than pleasure horses (*p* = 0.039 and *p* = 0.040, respectively) when started under saddle when older. Additionally, stallions were bolder than the reference group: geldings (*p* = 0.034). Brown horses and chestnut horses were less bold than the reference group: bay horses (*p* = 0.049 and *p* = 0.027, respectively). The details of these results are presented in Table 4.

### 3.3. Independence

Analysis of the coefficients revealed that horses started under saddle at an older age were less independent than horses started under saddle at an earlier age (*p* = 0.010). Furthermore, Australian Stock Horses were more independent than crossbred horses when started under saddle at an older age (*p* = 0.007), whereas Thoroughbreds were less independent (*p* = 0.000) than crossbred horses when started under saddle at an older age. Heavy horses (*p* = 0.029) and ponies (*p* = 0.044) were more independent than crossbred horses when started under saddle when older. Companion horses (*p* = 0.017) and horses used for mounted games (*p* = 0.033) were less independent than pleasure horses when started under saddle when older, whilst horses used for working equitation were more independent than pleasure horses (*p* = 0.020). Additionally, riders with more than eight years of riding experience reported having more independent horses than riders who had ridden their entire lives (*p* = 0.015). These results are presented in Table 5.

## 4. Discussion

The purpose of this study was to investigate age-related differences in ridden and unridden horse behaviour, as reported by horse owners and trainers through the E-BARQ. The results confirmed that boldness and independence are separate traits in horses and that there are some age-related differences associated with both of these traits. Boldness is a super-trait that is reported in many other species, including dogs and livestock. In equids, it is generally used as an umbrella term to describe individuals that are not shy, nervous or easily spooked. Independence, on the other hand, is less commonly reported in the literature than boldness but captures the individual’s ability to function without the social support of conspecifics and is therefore highly relevant to considerations of horse performance, horse welfare and rider/handler safety.

### 4.1. Boldness

The study confirmed that as a horse ages, behavioural traits associated with boldness increase. A possible explanation for this is that, with the accumulated exposure to various events and stimuli that come with time, horses generally become more habituated to novel stimuli and new environments, and amass more life experiences than their younger counterparts [48]. Likewise, such horses have undergone more training, exposing them to more stimuli from both riders and the immediate environment [48]. Our findings align with those of Baragli et al. [49], who reported that younger horses showed more frequent avoidance of an aversive stimulus than older horses did. In contrast, some studies conflict with the current results. Krueger et al. [50], König von Borstel et al. [51] and Gardner [37] found that younger horses were more exploratory than their older counterparts in non-social novel object testing. Their findings imply that younger horses are bolder than older horses. However, the horses in their studies were individually observed and scored for boldness by the experimenters rather than from scores based on owner-reported observations, as the current study did. The use of owner-reported observations may be considered a more accurate representation of boldness in horses than one-off tests results [52]. This may explain the difference among the studies.

In the current study, stallions were reported to be bolder than geldings. Bolder horses are thought to initiate more activity in herd settings [26]. This is generally the case for free-ranging stallions, in responding to a threat to their group, when they drive their harem mares to move forward from behind, thereby initiating movement away from the threat [26]. The stallions reported in the current study population reflect the boldness of their free-ranging counterparts. That said, it could also be that more experienced handlers are more likely to own stallions because they are better equipped to train and handle them than less experienced handlers. So, it is possible that the effect of experience of handlers may mean that these horses present as bolder.

The current results showed that the age at which horses were started under saddle differed across disciplines. This was expected. Horses in the current study started later were less bold than those started younger. Fundamentally, bolder horses may be started earlier than shy horses that may have been left until later in the hope that they may be calmer (and therefore safer) as they mature. Increased boldness could be explained by younger horses having had more training and habituation to stimuli specific to their disciplines before starting under saddle than horses with less training before starting under saddle. Therefore, these horses may be less reactive to novel stimuli. However, this trend was reversed in some breeds, such as the Australian Stock Horse, which was bolder when started under saddle later when compared to crossbreed horses. This could reflect breed differences in the breeds’ rates of development and emotional maturity. It certainly merits further exploration.

Australian Stock Horses were originally bred for the purpose of being sturdy saddle horses for explorers, stockman and settlers in the 18th Century [53]. This could explain why Australian Stock Horses in the current study were reported as being bolder. In comparison, crossbreed horses, particularly those with genetics of so-called excitable breeds (e.g., hot-blooded breeds including Thoroughbreds) [54], may be started late for reasons including early behavioural or temperament concerns requiring horses to undergo further pretraining and desensitisation before being saddled. Thus, when started late, Australian Stock Horses are likely to be bolder than crossbred horses.

Likewise, several disciplines were associated with increased boldness when started late when compared to pleasure horses. They were: breeding conformation (i.e., those used for exhibition based chiefly on their morphological characteristics), working equitation, eventing and working cow horses. Pleasure horses have been shown to have increased anxiety when compared to competition horses [55]. This could be attributed to the use of selective breeding for calmness in competition horses. Additionally, horses used in competitions are exposed more often to novel stimuli, thereby becoming habituated to them, when compared to pleasure horses. Those individuals that are unable to cope with stress are deemed undesirable for competition and may more commonly be used as leisure horses.

Ideally, horses used for showing must be safe around strangers (such as judges and stewards at shows) and those that go on to be used for breeding must be easy and safe to handle to ensure safety of personnel and conspecifics during breeding procedures (e.g., managed matings and semen collections). Horses that succeed in eventing must be agile, fast, bold and fearless [56]. Similarly, horses used in working equitation must be easy to train and relatively unreactive to novel stimuli or unfamiliar places, have low anxiety and be explorative [26]. Successful working cow horses are said to need good manners, smooth rideability, good cow sense and responsiveness to rein cues [57]. Working cow horses are often started younger, usually around 18 months of age to make them competitive for futurities at three years old [58]. Therefore, horses used for breeding conformation, working equitation, eventing and working cow horses are generally predisposed to increased boldness and are more likely to be bolder than pleasure horses when started late.

The current results show that decreased boldness was associated with a late start in dressage and therapy horses when compared to pleasure horses. This finding is consistent with previous reports that dressage horses were more fearful and emotional than horses used for show-jumping [23,59,60]. Hausberger et al. [60] found that, when led over an unknown obstacle (a bridge) by an unfamiliar experimenter, dressage horses showed more emotional reactions than show-jumpers. Horses used for therapeutic activities are often unwanted horses or those that have been retired from previous ridden careers [61], possibly due to behavioural issues or advancing age. Anderson et al. [62] assigned reactivity scores to college-owned horses (*n* = 22), privately owned horses (*n* = 8) and therapeutic riding program horses (*n* = 73). They found that 64% of horses with the highest reactivity scores (score of over 4 out of 5) were horses used in therapeutic riding programs. Horses with pre-existing behavioural issues entering therapeutic programs may not be appropriately desensitised to novel stimuli and environments. So, these programs may need to be more mindful of prospective horses’ temperaments at the point of selection [62]. Dressage and therapy horses may already be predisposed to decreased boldness and are therefore more likely to be less bold than pleasure horses when started late.

An additional finding from the current study was that brown and chestnut horses were less bold than bay horses. Although genes responsible for coat colour may be linked to quantitative trait loci for behavioural traits [63], few equine studies investigating correlations with coat colour and behaviour have been published [64,65]. However, with a smaller sample (*n* = 477) than the current one (*n* = 1940), Finn et al. [65] found that chestnut coloured horses were bolder than bay horses in that they were more likely to approach novel or unfamiliar objects and animals within their environment [65]. The current findings, as they relate to chestnut horses, contradict those of Finn et al. [65], and so further investigation into correlations between coat colour and boldness is warranted.

### 4.2. Independence

In the current study, independence was assessed according to horses’ behaviour when separated from conspecifics only and also when separated from both conspecifics and home. Unlike boldness, independence was not significantly associated with age. However, the results revealed that horses that had been started late were less independent than those started younger. Several factors could explain this, including the housing environment prior to starting under saddle that may have involved social isolation.

Increased independence associated with a late start was revealed in Australian Stock Horses, heavy horses and ponies when compared to crossbreed horses. As discussed earlier with reference to boldness, the nature of stock horse work may favour the selection of independent individuals that can adapt to social isolation and to unfamiliar environments. Draught horses are generally suitable for management in small stable groups because they are sometimes thought to become aggressive toward other individuals [66]. This prospect is supported by the report from Lloyd et al. [54], in which Irish Draught horses ranked lowest for sociability (along with American Quarter Horse) when compared to other breeds including Arabians and Thoroughbreds. Low sociability indicates that individuals are more self-reliant and better able to cope with isolation from conspecifics. Relatively small domestic equids, particularly Shetland ponies, Icelandic horses and Exmoor ponies, are often regarded as highly sociable and easy to manage in large groups [66]. In the current study, crossbreed horses may include those with bloodlines selected for high gregariousness and sociability. Subsequently, ponies may have become relatively less independent. Regardless, it can be assumed that being independent has benefits for rider safety. Sackman and Houpt [12] determined that ponies were the least nervous equids in their study when compared to other breeds including Thoroughbreds and Arabians. An explanation for this could be that ponies are generally ridden by children, and so are primarily selected for temperamental traits that advance safety [67]. So, independence is an important trait in equids intended for children and novice riders, in a way that boldness is not.

Likewise, when compared to pleasure horses, equids used for working equitation were more independent when started late. It can be speculated that horses used in working equitation must be independent in nature to remain focused in their work around competition equipment and obstacles and not become distracted in the presence of other horses or unfamiliar people or anxious in the absence of conspecifics.

Decreased independence, in the current study, was associated with a late start in Thoroughbreds when compared to crossbreed horses. McGreevy et al. [68] reported that Thoroughbreds were at high risk for stereotypic behaviour associated with social isolation from conspecifics, implying that Thoroughbreds do not cope well when separated from other horses or that they are isolated more than other breeds. Thoroughbreds are also thought to be more nervous and reactive than many other breeds [12,54,60]. Surprisingly, the current results suggest that starting Thoroughbreds under saddle early will produce more independent horses that are relatively easier to handle and train. As discussed for boldness, it could be speculated that, in the current study, horses started under saddle late had been held back because they were not independent when younger. So, trainers and owners may have elected to wait until these individuals were older in the hope that their independence would improve.

When compared to pleasure horses, companion horses and horses used for mounted games were less independent when started late. Companion horses are similar to pleasure horses and therapy horses in that generally they are not often specifically bred to be companions. Most often they are bred for a specific discipline (whether that be performance sports, stock work or another discipline) and are usually retired from their respective disciplines for a reason (including advancing age, behavioural issues, physical ailment or reduced athletic ability). Training is often delayed in behavioural cases, and horses may be sold multiple times before training begins. Horses with early behavioural issues often move homes regularly when young and are usually housed separately from familiar conspecifics or housed with few unfamiliar horses during traditional box and barn-weaning [69]. Such social flux can induce strong stress reactions in the naïve horse and is a risk factor for developing abnormal behaviours, including weaving [25,69]. Horses that are unable to participate in normal social behaviours are considered to have poor welfare, especially when coupled with abnormal behaviours [1,70].

Horses used for mounted games (including polo and polocrosse) are often housed in stalls but are in contact with other conspecifics, and usually compete alongside conspecifics. Mounted games are physically demanding for the horse [25] and so some horses may be started under saddle late to ensure adequate muscle growth, skeletal development and physical fitness before riding. The combination of social housing and regular conspecific and human interaction as the horses age could lead to their becoming less tolerable of social isolation. Thus, companion horses and horses used for mounted games may be relatively unaccustomed to social isolation and, as a result, may not cope as well as pleasure horses when away from conspecifics or away from home.

Finally, riders with over eight years’ riding experience reported having more independent horses than riders who had been riding their whole lives. Convention dictates that less experienced (or younger) riders are preferentially coupled with more experienced horses because these horses are generally safer as they have been exposed to more aversive stimuli throughout their lives [71]. Likewise, horses that are challenging to handle in the absence of other horses are usually considered unsuitable for inexperienced riders or leisure riders [72,73]. So, riders who have ridden their whole lives are expected to be better able to handle their horses and may report on relatively fewer independent horses in their care. Longitudinal research in this domain will help to unpick any causal effects.

### 4.3. Limitations

It is worth acknowledging some of the limitations of the current study. Fundamentally, the authors acknowledge that this survey allows only exploration of correlations between management and horse variables with the reported outcomes, rather than any causal relationships.

As with any online survey, the E-BARQ relies on self-reported data from respondents. Respondents were advised on the nature of the study via a participant information statement and then asked for their consent to participate in the survey. They were trusted to answer questions truthfully and to the best of their ability. Although the identities of respondents remained anonymous to the researchers, respondents were required to register with E-BARQ and provide personal information. Thus, although anonymity was guaranteed prior to undertaking the questionnaire, some respondents may have been disinclined to answer some questions truthfully due to social desirability or approval. This may have led to under-reporting of some unwelcome behaviours, such that the prevalence or extent of such outcomes may be worse than the current data suggest.

E-BARQ relies on respondents’ recall. It covers many behaviours, some of which can be infrequent, so respondents are asked to report on events and behaviours throughout the preceding six-month period. In previous studies, when asked to recall events within a three- to six-month period, respondents scored a three-out-of-five for recall, whereas respondents reporting events within a week of their occurrence scored a five-out-of-five for recall [54,74,75,76,77,78,79,80]. Previous investigations into the potential sources of bias inherent in equine behavioural data collection [52] revealed three main sources: recall, confirmation and sampling bias. As a validated instrument [46], E-BARQ attempts to minimise their impact.

With all data from online surveys, some caution must be applied when interpreting results, including the characteristics of non-respondents and difficulty contacting a range of respondents to truly represent the general population. That said, although non-respondent data are unavailable, the gender distribution of respondents in this E-BARQ is comparable to previous equine questionnaires. Women comprised the vast majority of respondents, similar to previous studies where the proportion of men ranged from 6 to 16% [14,33,81,82]. This indicates that women are more engaged in equitation than men. It is also possible that they are more likely than men to respond to surveys, potentially increasing respondent bias [33].

## 5. Conclusions

Behavioural traits in horses are important factors for owners and handlers when selecting and preparing horses for various disciplines. The current study confirms that boldness and independence are separate traits in horses but that only boldness increases with age. Generally, older horses were more likely to be bolder than younger horses, but horses started under saddle at a relatively older age were less bold and less independent. Boldness was positively associated with breed (Australian Stock Horses) and disciplines (breeding conformation, equitation, eventing and working cow horse) and negatively associated with dressage and therapy horses. Independence was positively associated with breed (Australian Stock Horses, heavy horses and ponies) and working equitation. Independence was also negatively associated with Thoroughbred horses and with companion horses and those used in mounted games. Additionally, stallions and coat colour (chestnut and brown) were positively associated with boldness while rider experience (eight years or more) was positively associated with independence. Understanding how age affects behavioural traits can improve horse–rider matching and projections of how young horses will mature behaviourally. Strategic use of this information has the potential to improve horse welfare.

## Figures and Tables

**Table 1:** Equine Behaviour Assessment and Research Questionnaire (E-BARQ) items in the five factors of interest for trait selection after rotation to facilitate interpretation. “T” represents the temperament factor from factor analysis (T1, T2, T11, T18, T20) and “RC” is the rotated component (RC1, RC2 and RC3). This parallel analysis suggested three underlying components with loadings as follows. Significant loadings are in bold.

Factor	Missing	Present	% Missing	RC1	RC2	RC3
Factor T1—Does your horse strongly avoid, shy or bolt away from:						
Bicycles	165	1836	8.25	**0.76**	0.11	0.06
Cars	164	1837	8.20	**0.77**	0.12	−0.08
Chainsaw	164	1837	8.20	**0.79**	0.13	0.01
Horse and cart	163	1838	8.15	**0.69**	0.13	0.15
Motorbikes	164	1837	8.20	**0.78**	0.13	−0.04
Umbrella	163	1838	8.15	**0.74**	0.14	0.13
Factor T2—When left alone, have you seen your horse:						
Box-walking	186	1815	9.30	0.08	**0.76**	0.2
Canter when alone	183	1818	9.15	0.13	**0.77**	0.09
Fence walking	184	1817	9.20	0.06	**0.57**	0.15
Pawing	187	1814	9.35	0.09	**0.69**	0.21
Sweating	188	1813	9.40%	0.11	**0.74**	0.04
Trembling	186	1815	9.30%	0.15	**0.66**	0.03
Vocalising	184	1817	9.20%	0.06	**0.73**	0.07
Factor T11—When left alone, have you seen your horse:						
Pulling back when alone	184	1817	9.20%	0.12	0.48	**0.66**
Pulling back when away from home	1304	697	65.17%	0.16	0.14	**0.86**
Standing still when away from home	1305	696	65.22%	0.17	0.31	**0.62**
Factor T18—Does your horse strongly avoid, shy or bolt away from:						
Plastic bags	164	1837	8.20%	**0.66**	0.08	0.17
Unfamiliar sights	112	1889	5.60%	**0.5**	0.19	0.15
Factor T20—Does your horse strongly avoid, shy or bolt away from:						
Children	164	1837	8.20%	**0.55**	−0.05	0.33
Dogs	163	1838	8.15%	0.48	−0.04	0.28

**Table 2:** Repeated parallel analysis with two T11 questions removed due to substantially lower response rates compared to other E-BARQ items. The number of rotated components was dropped from 3 to 2 from the previous analysis after suggestion to remove these questions. Significant loadings are in bold.

Factor	RC1	RC2
Factor T1		
Bicycles	**0.76**	0.11
Cars	**0.75**	0.08
Chainsaw	**0.78**	0.12
Horse and cart	**0.7**	0.16
Motorbikes	**0.76**	0.1
Umbrella	**0.75**	0.16
Factor T2		
Box-walking	0.09	**0.78**
Canter when alone	0.12	**0.77**
Fence walking	0.07	**0.6**
Pawing	0.1	**0.72**
Sweating	0.09	**0.73**
Trembling	0.13	**0.64**
Vocalising	0.05	**0.73**
Factor T11		
Pulling back when alone	0.19	**0.63**
Factor T18		
Plastic bags	**0.68**	0.12
Unfamiliar sights	**0.52**	0.22
Factor T20		
Children	**0.59**	0.04
Dogs	**0.52**	0.05

**Table 3:** Univariate analysis of other predictor variables (including potential confounders) for Boldness and Independence, assessed for potential inclusion by univariate analysis. Significant differences appear in bold.

Variable	Boldness		Independence	
	*f*-Value	*p*-Value	*f*-Value	*p*-Value
Gender of Rider	2.5715	0.077	2.3693	0.094
Country of Rider	1.4297	0.057	0.9587	0.534
Age of Rider	1.1022	0.359	2.4896	**0.011**
Laterality of Rider	1.4745	0.220	1.3352	0.261
Sex of Horse	2.8949	**0.013**	1.6406	0.161
Analysis colour	2.3817	**0.008**	2.4513	**0.007**
Height Horse	0.2968	**0.019**	0.7195	0.675
Analysis Breed	3.2396	**<0.001**	3.0623	**<0.001**
Rider Experience	1.2931	0.250	2.3489	**0.022**
Analysis Discipline	3.9995	**<0.001**	1.975	**0.006**

**Table 4:** Significant age-related variables for the suite of traits labelled Boldness for horses (*n* = 1940) reported by respondents in the E-BARQ.

Variable	Estimate	Std. Error	*t*-Value	*p*-Value
(Intercept) ^1^	−4.452	0.086	−51.742	0.000
Stallion	0.430	0.203	2.120	0.034
Colour of Horse (reference Bay)				
Brown	−0.167	0.085	−1.975	0.049
Chestnut	−0.167	0.065	−2.220	0.027
Age Parameters				
Age of horse	0.270	0.107	2.515	0.012
Age started under Saddle	−0.062	0.030	−2.060	0.040
Interaction of Age started under Saddle and Breed (reference crossbreed Horse)				
Age started under Saddle × Australian Stock Horse	0.164	0.066	2.469	0.014
Interaction of Age started under Saddle and Discipline (reference Pleasure Horse)				
Age started under Saddle × Breeding conformation	0.222	0.107	2.068	0.039
Age started under Saddle × Dressage	−0.055	0.026	−2.072	0.039
Age started under Saddle × Working Equitation	0.154	0.077	2.010	0.045
Age started under Saddle × Eventing	0.072	0.036	2.017	0.044
Age started under Saddle × Therapy horse	−0.486	0.236	−2.062	0.040
Age started under Saddle × Working cow horse	0.211	0.099	2.125	0.034

^1^ The expected value of the dependent variable.

**Table 5:** Significant age-related variables (in bold) for the suite of traits labelled Independence for horses (*n* = 1940) reported by respondents in the E-BARQ.

Variable	Estimate	Std. Error	*t*-Value	*p*-Value
(Intercept) ^1^	−4.161	0.105	−39.717	**0.000**
Rider Experience (reference I’ve ridden horses all my life)				
>8 years’ experience	0.171	0.070	2.447	**0.015**
Age Parameters				
Age of horse	−0.001	0.004	−0.165	0.869
Age started under Saddle	−0.080	0.031	−2.574	**0.010**
Interaction of Age started under saddle and breed (reference crossbreed horse)				
Age started under Saddle × Australian Stock Horse	0.179	0.067	2.688	**0.007**
Age started under Saddle × Thoroughbred	−0.169	0.043	−3.933	**0.000**
Age started under Saddle × Heavy Horse	0.100	0.046	2.192	**0.029**
Age started under Saddle × Pony	0.103	0.051	2.013	**0.044**
Interaction of Age started under saddle and discipline (reference pleasure horse)				
Age started under Saddle × Companion horse	−0.160	0.067	−2.391	**0.017**
Age started under Saddle × Working equitation	0.180	0.077	2.331	**0.020**
Age started under Saddle × Mounted games	−0.180	0.084	−2.132	**0.033**

^1^ The expected value of the dependent variable.

## Supplementary Materials

The following are available online at https://www.mdpi.com/2076-2615/10/12/2321/s1.
