# Supplementary material for: Age-Related Changes in the Behaviour of Domestic Horses as Reported by Owners
Source: Animals (Basel). 2020 Dec 7;10(12):2321. doi: 10.3390/ani10122321 (PMC7762420; doi:10.3390/ani10122321)
Supplement: Supplementary file 1 [file animals-10-02321-s001.pdf]

English

PIS

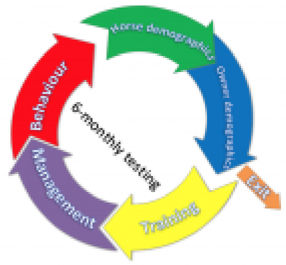

The E-BARQ questionnaire will take approximately 20 - 30 minutes to complete.
E-BARQ is voluntary and your information is confidential.
If you answer all of the questions, you will receive a ***Share-&-Compare*** graph on completion. This graph will show you where your horse compares to the population on 14 different categories, including Trainability, Rideability, Social Confidence and so on. Please **respond to all questions** to receive your graph (which can be found on your **E-BARQ dashboard** (under the E-BARQ Results tab) , immediately on completion).
Please click here to download the E-BARQ personal information statement.

I have read and agreed to the Personal Information Statement and Terms and Conditions of the E-BARQ project.

Yes

No (this option will remove you from E-BARQ)

Your email address registered: **${e://Field/user}**

Is this your FIRST time completing an E-BARQ questionnaire?
Select 'No' if you already have an E-BARQ Dashboard (have completed an E-BARQ for another horse).

Yes

No, I have completed an E-BARQ previously

1st E-BARQ Demographics

Are you?

In which country do you reside?

What is your age?

Are you RIGHT or LEFT handed?

Demographics

Your horse's name: ${e://Field/horsename}
Your horse's E-BARQ ID: ${e://Field/ebarqid}

You are welcome to complete one E-BARQ for each horse that you own but this survey will refer only to the horse named here.

What sex is ${e://Field/horsename}?

Has ${e://Field/horsename} ever covered a mare?

Yes

No

I don't have this information

At approximately what age was ${e://Field/horsename} gelded?

What was the MAIN reason ${e://Field/horsename} was gelded?

Has ${e://Field/horsename} ever had a foal?

Is ${e://Field/horsename} any different to handle when in season?

How old is ${e://Field/horsename} in years?
Please use a whole number or a decimal for less than one year (6 months = 0.5)

Age in years

Is ${e://Field/horsename} any different to ride/drive when in season?

Has ${e://Field/horsename} ever been on an Altrenogest hormonal treatment such as Regu-Mate, Readyserve, Ovu-Mate or Matrix?

What color is ${e://Field/horsename}?

How tall is ${e://Field/horsename}?

Is ${e://Field/horsename} pure-bred?

What breed is ${e://Field/horsename}?

You have told us that ${e://Field/horsename} is not a pure-bred horse, can you now tell us about ${e://Field/horsename}'s parentage?

Choose up to 4 breeds that contribute to ${q://QID11/ChoiceTextEntryValue/4}*'s genetic makeup.*

Abyssinian

Akhal Teke

Albanian

Altai

American Cream Draft

American Crème and White

American Walking Pony

Andalusian

Andravida

Anglo-Kabarda

Appaloosa

Araappaloosa

Arabian

Ardennes

Argentine Criollo

Asturian

Australian Brumby

Australian Pony

Australian Stock Horse

Azteca

Balearic

Baluchi

Ban-ei

Banker

Barb

Bashkir

Bashkir Curly

Basotho Pony

Belgian Warmblood

Belgian

Bhirum Pony

Bhotia Pony

Boer

Bosnian Mountain Horse

Breton

Brumby

Budyonny

Byelorussian Harness Horse

Camargue

Campolina

Canadian

Carthusian

Caspian

Cayuse Indian Pony

Cheju

Chilean Corralero

Chincoteague Pony

Cleveland Bay

Clydesdale

Colorado Ranger Horse

Connemara Pony

Criollo (Uruguay)

Crioulo

Dales Pony

Danish Warmblood

Danube

Dartmoor Pony

Deliboz

Djerma

DØle

Dongola

Dülmen Pony

Dutch Draft

Dutch Warmblood

East Bulgarian

Egyptian

Eriskay Pony

Estonian Native

Exmoor Pony

Faeroes Pony

Falabella

Fell Pony

Finnhorse

Fleuve

Florida Cracker Horse

Fouta

Frederiksborg

French Saddlebred

French Trotter

Friesian

Galiceño

Galician Pony

Gelderlander

German Riding Pony

Gidran

Golden American Saddlebred

Gotland

Groningen

Guangxi

Gypsy Cob

Hackney

Haflinger

Hanoverian

Hequ

Highland Pony

Hokkaido

Holsteiner

Hucul

Hungarian Warmblood

Iberian

Icelandic

Iomud

Irish Draft

Irish Sport Horse

Jinzhou

Jutland

Kabarda

Karabair

Karabakh

Kathiawari

Kazakh

Kerry Bog Pony

Kiger Mustang

Kirdi Pony

Kisber Felver

Kiso

Kladruby

Knabstrup

Kushum

Kustanai

Latvian

Lipizzan

Lithuanian Heavy Draft

Lokai

Losino

Lusitano

M'Bayar

Malopolski

Mangalarga

Marwari

Messara Pony

Miniature

Misaki

Missouri Fox Trotting Horse

Miyako

Mongolian

Morab

Morgan

Moyle

Mule

Murgese

Mustang

National Show Horse

New Forest Pony

New Kirgiz

New Zealand Kaimanawa

Newfoundland Pony

Nokota

Noma

Nooitgedacht Pony

Noric

Norland

North Swedish Horse

Northeastern

Norwegian Fjord

Ob

Oldenburg

Orlov Trotter

Paint

Pantaneiro

Paso Fino

Percheron

Peruvian Paso

Pindos Pony

Pinia

Pintabian

Piquira

Polish Konik

Pony of the Americas

Pottok

Przewalski

Pyrenean Tarpan

Qatgani

Quarab

Quarter Horse

Quarter Pony

Racking Horse

Rocky Mountain Horse

Russian Don

Russian Heavy Draft

Russian Trotter

Saddlebred

Sanhe

Schleswiger Heavy Draft

Schwarzälder Fuchs

Selle Francais

Shagya

Shetland Pony

Shire

Single-Footing Horse

Skyros Pony

Somali Pony

Sorraia

Soviet Heavy Draft

Spanish Mustang

Spanish Norman

Spanish-Barb

Standardbred

Sudan Country-Bred

Suffolk

Swedish Warmblood

Taishuh

Tawleed

Tennessee Walking Horse

Tersk

Thessalian

Thoroughbred

Tokara

Tori

Trakehner

Ukraninian Saddle Horse

Vlaamperd

Vladimir Heavy Draft

Vyatka

Waler

Warlander

Welara Pony

Welsh Cob

Welsh Pony

West African Barb

Western Sudan Pony

Wielkopolski

Xilingol

Yakut

Yanqi

Yili

Yonaguni

Zaniskari Pony

Zhemaich

Unknown

Other

Which best describes ${e://Field/horsename}'s history?

Where did you acquire ${e://Field/horsename}?

Approximately how long has ${e://Field/horsename} been with you?

How old was this horse when you acquired it?

Approximately how old was ${e://Field/horsename} when handling started?

General handling, including leading and grooming.

When was ${e://Field/horsename} started under saddle or in harness?

Here, starting under saddle means to carry a rider, not simply to wear a saddle and in harness means to be attached to a vehicle.

Have you had a saddle professionally fitted to ${e://Field/horsename} in the past 6 months?

Have you found ${e://Field/horsename} difficult to fit a saddle to?
If so, choose as many of the following as you feel are appropriate.
${e://Field/horsename} is:

This horse is not difficult to fit a saddle to

Very fat

High withered

Flat or mutton withered

Naturally very wide

Short backed

Long backed

Suffering from an injury of the back or withers

Only able to wear a bareback pad

I'm not sure but the horse seems uncomfortable wearing a saddle

Other behavioral reason

Other conformational reason

Other veterinary reason

Which statement best describes ${e://Field/horsename}'s diet?

Is this the first horse you have owned or worked with regularly?

Did you keep or work with horses as a child (before the age of 16)?

Do you have any other horses at the moment?

Do you wear a safety helmet when:

	Never	Rarely	Sometimes	Usually	Always	N
Riding/driving your horse	○	○	○	○	○	○

	Never	Rarely	Sometimes	Usually	Always	N
Working your horse on the ground	○	○	○	○	○	(
For general ground handling	○	○	○	○	○	(

Are you currently working in the horse industry?

Check as many as apply.

No

Trainer

Instructor/coach

Paid rider/jockey/driver

Groom/stable hand

Boarding facility/livery/agistment owner

Rescue organization

Equine-assisted therapy worker

Veterinarian

Equine researcher

Teacher/lecturer

Vet nurse, technician or assistant

Behavior consultant

Nutritionist

Equine body-worker (e.g. massage, chiropractic)

Farrier/blacksmith/barefoot trimmer

Other

Select the option that best describes your experience with horses:

Using the previous 12 months as a guide, how many different horses do you ride or handle per month?

Using the past 12 months as a guide, how many different handlers/riders are likely to ride or handle ${e://Field/horsename} per month?

I consider myself to be:

What is ${e://Field/horsename}'s MAIN discipline?

Has ${e://Field/horsename}:

	No	Yes	I don't know
Ever been trained by a professional	○	○	○
Had a serious accident or trauma	○	○	○
Ever been abused or had to be 'rescued'	○	○	○
Often been aggressive to other horses in the paddock	○	○	○
Often been attacked by other horses in the paddock	○	○	○

Is ${e://Field/horsename} currently suffering from an injury or illness?

Has ${e://Field/horsename} been assessed by a professional (equine behaviorist or veterinarian) for behavioral problems?

Has ${e://Field/horsename} been prescribed or given (over the counter) behavior-controlling supplements or medications?

Has ${e://Field/horsename} been ridden/driven in the last 6 months?

Yes

No

Have you, over the previous 6 months, experienced any problems with ${e://Field/horsename}'s behavior or temperament?

No

Only minor problems

Moderate problems

Serious problems

Non-Ridden

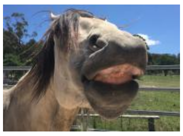 We understand that the survey is long and very much appreciate your time!

During the last 6 months, how often has ${e://Field/horsename}?

	Never	1-2 times	3-6 times	Once a month	Once a fortnight	W[illegible]
Travelled in a float/trailer	○	○	○	○	○	

	Never	1-2 times	3-6 times	Once a month	Once a fortnight	W[illegible]
Been to a show/competition	○	○	○	○	○	
Done ground/in-hand work	○	○	○	○	○	
Done round pen, loose schooling or liberty work at speed (trot or faster)	○	○	○	○	○	
Done clicker training or liberty work at walk	○	○	○	○	○	
Been lunged	○	○	○	○	○	
Been long-reined	○	○	○	○	○	
Been handled by professional horse people	○	○	○	○	○	
Been handled by teenagers	○	○	○	○	○	
Been handled by children	○	○	○	○	○	
Been handled by men/boys	○	○	○	○	○	

	Never	1-2 times	3-6 times	Once a month	Once a fortnight	W[illegible]
Been handled by women/girls	○	○	○	○	○	
Been trained using food rewards	○	○	○	○	○	

What has been your MAIN reason for spending time with ${e://Field/horsename} during the previous 6 months?

How is ${e://Field/horsename} housed during the SUMMER months of the year? Or the WET Season for those horses in the Tropics?

How is ${e://Field/horsename} housed during the WINTER months of the year?
Or the DRY Season for those horses in the Tropics?

Will ${e://Field/horsename} stand (when restrained only by a head collar and lead rope) for?
0 = never stands still, 4 = always stands still

	Never	1	2	3	4 - Always	Not observed/ applicable
General examination by veterinarian	○	○	○	○	○	○
Teeth examined by dentist/veterinarian	○	○	○	○	○	○
Hooves cleaned	○	○	○	○	○	○
Hooves trimmed	○	○	○	○	○	○
Shoeing	○	○	○	○	○	○

Does ${e://Field/horsename}?

						N[illegible] ob[illegible]

	Never	Rarely	Sometimes	Usually	Always	ap N
	Never	Rarely	Sometimes	Usually	Always	ob ap
Get distracted easily by unfamiliar sights	○	○	○	○	○	
Get distracted easily by unfamiliar sounds	○	○	○	○	○	
Have to be lunged, long-reined, worked on-line, loose schooled or round penned before riding/driving	○	○	○	○	○	
Back when signaled to move forward	○	○	○	○	○	
Not move when signaled	○	○	○	○	○	
Rear when signaled to go forward	○	○	○	○	○	

	Never	Rarely	Sometimes	Usually	Always	N[illegible] ob[illegible] ap[illegible]
Slow when signaled to go faster						
Fail to slow when signaled by a rein or lead rope cue						
Fail to stop when signaled by a rein or lead rope cue						
Raise head to avoid rein or lead rope cues						
Toss head when led						
Swish tail during work						
Pull on reins or lead rope when signals are applied						

	Never	Rarely	Sometimes	Usually	Always	N ob ap
Brace neck when rein or lead rope signals are applied						
Pick up the correct lead when signaled to canter LEFT on the ground (lunging/at liberty)						
Pick up the correct lead when signaled to canter RIGHT on the ground (lunging/at liberty)						
Buck, pigroot or kick out when signaled to canter when loose schooled or lunged						
Rear up when led						

	Never	Rarely	Sometimes	Usually	Always	N[illegible] ob[illegible] ap[illegible]
Rear up and flip over at any time	○	○	○	○	○	

During the past 6 months, when necessary before riding/driving, ${e://Field/horsename} has usually been:

The pre-ride/pre-drive lunging or schooling usually takes:

Estimate an average for the past 6 months

When being led or worked on the ground, ${e://Field/horsename} is responsive to:

	Strongly agree	Agree	Neutral	Disagree	Strongly disagree	No obs[illegible] app[illegible]

Voice cues to increase speed	Strongly agree	Agree	Neutral	Disagree	Strongly disagree	No obs app
Voice cues to decrease speed	○	○	○	○	○	
Voice cues to change gait	○	○	○	○	○	
Lead rope or rein tension to turn	○	○	○	○	○	
Lead rope or rein tension to slow from walk to halt	○	○	○	○	○	
Lead rope or rein tension to slow from trot to walk	○	○	○	○	○	

	Strongly agree	Agree	Neutral	Disagree	Strongly disagree	No obs app
Rein tension to slow from canter to trot	◯	◯	◯	◯	◯	
Whip application (with contact)	◯	◯	◯	◯	◯	
Whip cue (without contact)	◯	◯	◯	◯	◯	

When on a lead rope in a familiar or typical situation, does ${e://Field/horsename} pull:

	Never	Rarely	Sometimes	Usually	Always	Not observ applica
Forward when walking	◯	◯	◯	◯	◯	◯
Forward when trotting	◯	◯	◯	◯	◯	◯

Behind (lagging)	Never	Rarely	Sometimes	Usually	Always	Not observ applica
Forward when signaled to stop	◯	◯	◯	◯	◯	◯

When teaching a new lesson, ${e://Field/horsename} learns quickly?

	Strongly agree	Agree	Neutral	Disagree	Strongly disagree
With food rewards	◯	◯	◯	◯	◯
With positive reinforcement (other than food, eg. neck scratch)	◯	◯	◯	◯	◯
With pressure-release	◯	◯	◯	◯	◯
When corrected/punished	◯	◯	◯	◯	◯

During the past 6 months, has ${e://Field/horsename} been afraid of the following?

	Never	Rarely	Sometimes	Usually	Always
Bicycles	○	○	○	○	○
Motor bikes, quad bikes, all-terrain vehicles or tractors	○	○	○	○	○
Cars, trucks or trailers	○	○	○	○	○
Horse drawn vehicles	○	○	○	○	○
Chain saws	○	○	○	○	○
Lawn mowers	○	○	○	○	○
Umbrellas	○	○	○	○	○
Tents	○	○	○	○	○
Plastic bags	○	○	○	○	○
Strollers/prams	○	○	○	○	○
Children	○	○	○	○	○

	Never	Rarely	Sometimes	Usually	Always
Dogs	○	○	○	○	○
Wild animals (deer, kangaroo)	○	○	○	○	○
Domestic animals (cow, sheep, chickens)	○	○	○	○	○
Uncommon domestic animals (alpacas, pigs)	○	○	○	○	○

Some horses display **defensive** or **aggressive** behavior in certain situations.
Typical **moderate signs** would include **threatening** to *bite*, *pinning ears*, *tail swishing*, threatening to *kick* or *strike*. The most **serious signs** would include **actual** *biting*, *kicking* or *striking*.
Check a box on the 5-point scale below to indicate ${q://QID11/ChoiceTextEntryValue/4}'s recent tendency (using the past 6 months as a guide) to show these behaviors in the following context:

	1 - No signs	2	3	4	5 - Serious signs (kick, bite, strike)	Not observed/ applicable

	1 - No signs	2	3	4	5 - Serious signs (kick, bite, strike)	Not observed/ applicable
When verbally corrected by you or another person on the ground	○	○	○	○	○	○
When approached by you in the paddock	○	○	○	○	○	○
When approached by you when tied/tethered	○	○	○	○	○	○
When approached by you in the stable/stall	○	○	○	○	○	○
When approached by an unfamiliar person in the paddock or stable/stall or small enclosure	○	○	○	○	○	○

	1 - No signs	2	3	4	5 - Serious signs (kick, bite, strike)	Not observed/ applicable
When approached by an unfamiliar person when tied/tethered	○	○	○	○	○	○
When approached by you when eating from a bucket or manger	○	○	○	○	○	○
When approached by you carrying a bucket of feed	○	○	○	○	○	○
When a roller/surcingle is placed on his/her back	○	○	○	○	○	○
When the girth is done up	○	○	○	○	○	○
When is hosed down	○	○	○	○	○	○
When approached by a familiar dog	○	○	○	○	○	○

	1 - No signs	2	3	4	5 - Serious signs (kick, bite, strike)	Not observed/ applicable
When approached by a familiar animal of another species (goat, cat, rabbit, etc.)	○	○	○	○	○	○
When approached by an unfamiliar dog	○	○	○	○	○	○
When approached by an unfamiliar animal of another species (goat, cat, rabbit, etc.)	○	○	○	○	○	○
When being led towards an unfamiliar horse	○	○	○	○	○	○
When approached by an unfamiliar horse in the stall/stable or paddock/pasture	○	○	○	○	○	○

	1 - No signs	2	3	4	5 - Serious signs (kick, bite, strike)	Not observed/ applicable
When being led beside an unfamiliar horse	○	○	○	○	○	○
When being lunged or worked in a round pen	○	○	○	○	○	○
When signaled to go forward when on the lead (or lead rope)	○	○	○	○	○	○
When signaled to canter	○	○	○	○	○	○
When signaled to make an upwards transition	○	○	○	○	○	○

Some horses show signs of anxiety when separated from other horses. Thinking back (over the past 6 months as a reference), how often does ${e://Field/horsename} show signs of each of the following behaviors when separated from other horses?

	Never	Rarely	Sometimes	Usually	Always	I don't know
Trembling	○	○	○	○	○	○
Sweating	○	○	○	○	○	○
Trotting, cantering or galloping in the field	○	○	○	○	○	○
Jumping or pushing through a gate or fence	○	○	○	○	○	○
Pacing, circling, rearing etc, in the stall	○	○	○	○	○	○
Vocalizing (calling out or neighing)	○	○	○	○	○	○
Pawing	○	○	○	○	○	○

[illegible]

Pulling back when tied	Never	Rarely	Sometimes	Usually	Always	I don't know

Does ${e://Field/horsename} show more anxiety when:

Some horses show signs of anxiety when away from home. Thinking back (over the past 6 months as a reference), how often does ${e://Field/horsename} show signs of each of the following behaviors when away from home (either when led or driven to a new location)?

	Never	Rarely	Sometimes	Usually	Always	Nc ob Ap
Restlessness	○	○	○	○	○	
Pacing	○	○	○	○	○	
Pulling at [illegible]	○	○	○	○	○	

	Never	Rarely	Sometimes	Usually	Always	Nc ob Ap
the lead or reins	○	○	○	○	○	
Vocalizing	○	○	○	○	○	
Bucking	○	○	○	○	○	
Rearing	○	○	○	○	○	
Bolting	○	○	○	○	○	
Pulling back when tied	○	○	○	○	○	
Moving about when tied	○	○	○	○	○	
Other escape behaviors	○	○	○	○	○	

How often does ${e://Field/horsename}?

	Never	Rarely	Sometimes	Usually	Always	D kı
Wind-suck or crib-bite	○	○	○	○	○	(

	Never	Rarely	Sometimes	Usually	Always	D k[illegible]
Weave						
Chew own rugs/blankets						
Chew other horses' rugs/blankets						
Chew wood (fence or stable)						
Chew bark						
Eat feces						
Eat soil						
Dunk hay in water						
Play with water						
Paw prior to feeding						
Paw when feeding						

	Never	Rarely	Sometimes	Usually	Always	D kı
Hold up one foot when feeding	○	○	○	○	○	(
Kick out with a hind foot while eating	○	○	○	○	○	(
Show signs of enjoying grooming (such as extending the neck or wiggling the upper lip)	○	○	○	○	○	(
Unfasten gates	○	○	○	○	○	(
Push gates open	○	○	○	○	○	(
Box/stall walk	○	○	○	○	○	(
Walk the fence line repeatedly	○	○	○	○	○	(
Head shake (side to side)	○	○	○	○	○	(

	Never	Rarely	Sometimes	Usually	Always	D k[illegible]
Head toss (up and down)						
Groan when being worked						
Have its nose behind the vertical when lunged or long-reined						
Yawn before, during or after work						
Respond with over-sensitivity to touch						
Lie down during work or handling						
Fall down in the stall						

In the past 6 months, apart from when you are there, how frequently has ${e://Field/horsename} been in the company of other horses (where they can touch and groom one another), whether in the paddock, field or housed indoors?

Thinking back over the past 6 months, how many times has ${e://Field/horsename}?

	Never	1 Time	2 Times	3 Times	4 Times	5 or Mor Tim
Had a dental examination by a horse dentist or veterinarian	○	○	○	○	○	○
Been examined by a veterinarian for any reason (other than vaccinations or routine dental care)	○	○	○	○	○	○
Had body-work (chiropractic, massage etc.) done	○	○	○	○	○	○
Been shod	○	○	○	○	○	○
Had feet trimmed by a professional	○	○	○	○	○	○

	Never	1 Time	2 Times	3 Times	4 Times	5 or Mor[illegible] Tim[illegible]
Had feet trimmed by a non-professional	○	○	○	○	○	○
Been wormed/dewormed (with a commercial wormer such as a paste, drench, pellets)	○	○	○	○	○	○

Has ${e://Field/horsename} been vaccinated against Tetanus in the last 12 months?

Check 'yes' if boosters are up to date but 'no' if it's been 12 months or longer since last booster, even if it's booked in/scheduled.

Is ${e://Field/horsename} regularly vaccinated against any other diseases?

Such as Strangles, Hendra, West Nile etc.

Does ${e://Field/horsename}?

	Never	Rarely	Sometimes	Usually	Always
Come when called in the field	○	○	○	○	○
Avoid being caught (move away)	○	○	○	○	○
Pull back when tied	○	○	○	○	○
Throw head up when being bridled/haltered	○	○	○	○	○
Step back when being bridled/haltered	○	○	○	○	○
Throw head up when bridle/halter removed	○	○	○	○	○
Step back when bridled/halter removed	○	○	○	○	○

	Never	Rarely	Sometimes	Usually	Always
Load easily/without resistance onto the trailer	○	○	○	○	○
Load easily/without resistance onto the truck	○	○	○	○	○
Unload slowly off the trailer	○	○	○	○	○
Rush off the trailer backwards when loading	○	○	○	○	○
Rush off the trailer backwards when unloading	○	○	○	○	○
Rush on to the trailer forwards when loading	○	○	○	○	○
Unload slowly off the truck	○	○	○	○	○
Fall when travelling in the trailer	○	○	○	○	○

	Never	Rarely	Sometimes	Usually	Always
Kick when travelling in the trailer	○	○	○	○	○
Vocalize when travelling in the trailer	○	○	○	○	○
Walk into handler when led	○	○	○	○	○
Push handler when offered food	○	○	○	○	○

How often have you spent time with ${e://Field/horsename} over the past 6 months?

Include any activity 15 minutes or longer - grooming, feeding, visiting, petting, riding, exercising and ground work.

Ridden in the past 6 months

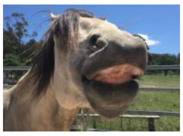 We understand that the survey is long and very much appreciate your time!

During the past 6 months, how often has ${e://Field/horsename}?

	Never	1-2 times	3-6 times	Once a month	Once a fortnight	W
Been ridden/driven in a lesson	○	○	○	○	○	
Travelled in a float/trailer	○	○	○	○	○	
Been to a show/competition	○	○	○	○	○	
Gone on a trail ride (or in harness on the trail)	○	○	○	○	○	
Been trained in an arena	○	○	○	○	○	

	Never	1-2 times	3-6 times	Once a month	Once a fortnight	W
Done ground/in-hand work						
Done round pen, loose schooling or liberty work at speed (trot or faster)						
Done clicker training or liberty work at walk						
Been lunged						
Been long-reined						
Been ridden/driven by beginners or novice riders (less than 1 year of experience)						
Been ridden/driven by professionals						
Been ridden/driven by teenagers						

	Never	1-2 times	3-6 times	Once a month	Once a fortnight	W[illegible]
Been ridden/driven by children	◯	◯	◯	◯	◯	
Been ridden/driven by men/boys	◯	◯	◯	◯	◯	
Been ridden/driven by women/girls	◯	◯	◯	◯	◯	
Been trained using food rewards	◯	◯	◯	◯	◯	

What has been your MAIN reason for riding/driving ${e://Field/horsename} during the previous 6 months?

How is ${e://Field/horsename} housed during the SUMMER months of the year?
Or the WET Season for those horses in the Tropics?

How is ${e://Field/horsename} housed during the WINTER months of the year?
Or the DRY Season for those horses in the Tropics?

Will ${e://Field/horsename} stand (when restrained only by a head collar and lead rope) for?
0 = never stands still, 4 = always stands still

	Never	1	2	3	4 - Always	Not observed/ applicable
General examination by veterinarian	○	○	○	○	○	○
Teeth examined by dentist/veterinarian	○	○	○	○	○	○
Hooves cleaned	○	○	○	○	○	○
Hooves trimmed	○	○	○	○	○	○

Shoeing	○	○	○	○	○	○
	Never	1	2	3	4 - Always	Not observed/ applicable

Does ${e://Field/horsename}?

	Never	Rarely	Sometimes	Usually	Always
Get distracted easily by unfamiliar sights	○	○	○	○	○
Get distracted easily by unfamiliar sounds	○	○	○	○	○
Have to be lunged, long-reined, worked on-line, loose schooled or round penned before riding/driving	○	○	○	○	○
Move off while you are mounting	○	○	○	○	○

	Never	Rarely	Sometimes	Usually	Always
Back when signaled to move forward	◯	◯	◯	◯	◯
Not move when signaled with leg or whip cues	◯	◯	◯	◯	◯
Rear when signaled to go forward	◯	◯	◯	◯	◯
Slow when signaled to go faster	◯	◯	◯	◯	◯
Fail to slow when signaled by a rein or lead rope cue	◯	◯	◯	◯	◯
Fail to stop when signaled by a rein or lead rope cue	◯	◯	◯	◯	◯
Fail to maintain speed or gait	◯	◯	◯	◯	◯

	Never	Rarely	Sometimes	Usually	Always	[illegible]
Need repeated steering to remain on a set course or line	○	○	○	○	○	
Bolt (gallop uncontrollably)	○	○	○	○	○	
Raise head to avoid rein or lead rope cues	○	○	○	○	○	
Toss head when being ridden/driven	○	○	○	○	○	
Swish tail during work	○	○	○	○	○	
Pull on reins or lead rope when signals are applied	○	○	○	○	○	
Brace neck when rein or lead rope signals are applied	○	○	○	○	○	

	Never	Rarely	Sometimes	Usually	Always
Move faster or raise head when anticipating the transition to canter	◯	◯	◯	◯	◯
Pick up the correct lead when signaled to canter to the LEFT under saddle	◯	◯	◯	◯	◯
Pick up the correct lead when signaled to canter to the RIGHT under saddle	◯	◯	◯	◯	◯
Pick up the correct lead when signaled to canter LEFT on the ground (lunging/at liberty)	◯	◯	◯	◯	◯
Pick up the correct lead when signaled to canter RIGHT on the ground (lunging/at liberty)	◯	◯	◯	◯	◯

	Never	Rarely	Sometimes	Usually	Always
Buck, pigroot or kick out when signaled to canter	○	○	○	○	○
Buck at other times (when ridden)	○	○	○	○	○
Unseat rider when bucking, pigrooting or kicking out	○	○	○	○	○
Rear up under saddle	○	○	○	○	○
Rear up and flip over at any time	○	○	○	○	○

During the past 6 months, when necessary before riding/driving, ${e://Field/horsename} has usually been:

The pre-ride/pre-drive lunging or schooling usually takes:

Estimate an average for the past 6 months

When ridden or driven, ${e://Field/horsename} is responsive to:

	Strongly agree	Agree	Neutral	Disagree	Strongly disagree	No obs app
Voice cues to increase speed	○	○	○	○	○	
Voice cues to decrease speed	○	○	○	○	○	
Voice cues to change gait	○	○	○	○	○	
Leg pressure to go from walk to trot	○	○	○	○	○	

	Strongly agree	Agree	Neutral	Disagree	Strongly disagree	No obs app
Leg pressure to go from trot to canter	◯	◯	◯	◯	◯	
Leg pressure to go from walk to canter	◯	◯	◯	◯	◯	
Leg pressure to go from halt to trot	◯	◯	◯	◯	◯	
Leg pressure to go from halt to canter	◯	◯	◯	◯	◯	
Rein tension to turn	◯	◯	◯	◯	◯	
Neck reining to turn	◯	◯	◯	◯	◯	

	Strongly agree	Agree	Neutral	Disagree	Strongly disagree	No obs app
Rein tension to slow from walk to halt	◯	◯	◯	◯	◯	
Rein tension to slow from canter to trot	◯	◯	◯	◯	◯	
Seat cues for upward transitions	◯	◯	◯	◯	◯	
Seat cues for downward transitions	◯	◯	◯	◯	◯	
Whip application (with contact)	◯	◯	◯	◯	◯	
Whip cue (without contact)	◯	◯	◯	◯	◯	

When on a lead rope in a familiar or typical situation, does ${e://Field/horsename} pull:

	Never Never	Rarely Rarely	Sometimes Sometimes	Usually Usually	Always Always	Not Not observ observ applica applica
Forward when walking	○	○	○	○	○	○
Forward when trotting	○	○	○	○	○	○
Behind (lagging)	○	○	○	○	○	○
Forward when signaled to stop	○	○	○	○	○	○

When teaching a new lesson, ${e://Field/horsename} learns quickly?

	Strongly agree	Agree	Neutral	Disagree	Strongly disagree
With food rewards	○	○	○	○	○

	Strongly agree	Agree	Neutral	Disagree	Strongly disagree
With positive reinforcement (other than food, eg. neck scratch)	○	○	○	○	○
With pressure-release	○	○	○	○	○
When corrected/punished	○	○	○	○	○

When ridden during the past 6 months, has ${e://Field/horsename} worn?

	Never	Rarely	Sometimes	Usually	Always	I don[illegible] kno[illegible] wha[illegible] that is
An English saddle	○	○	○	○	○	○
A Western or Australian stock saddle	○	○	○	○	○	○

	Never	Rarely	Sometimes	Usually	Always	I don[illegible] kno[illegible] wha[illegible] that is
A Treeless saddle	○	○	○	○	○	○
A bareback pad	○	○	○	○	○	○
No saddle	○	○	○	○	○	○
A snaffle bit (including multi-jointed, eggbutt, full-cheek and so on)	○	○	○	○	○	○
An English curb bit (including Weymouth, Pelham, Kimberwick and so on)	○	○	○	○	○	○
A Western curb (shanked) bit	○	○	○	○	○	○
A gag bit	○	○	○	○	○	○

A bitless bridle	Never	Rarely	Sometimes	Usually	Always	I don kno wha that is
Micklem bridle	○	○	○	○	○	○
A halter for riding	○	○	○	○	○	○
No bridle or halter for riding	○	○	○	○	○	○
A cavesson noseband	○	○	○	○	○	○
A drop noseband	○	○	○	○	○	○
A crank (Swedish) noseband	○	○	○	○	○	○
A grackle (crossed or figure 8) noseband	○	○	○	○	○	○
A double bridle	○	○	○	○	○	○

	Never	Rarely	Sometimes	Usually	Always	I don[illegible] kno[illegible] wha[illegible] that[illegible] is
No noseband for riding	○	○	○	○	○	○
A running or standing martingale	○	○	○	○	○	○
Side reins (for lunging or riding)	○	○	○	○	○	○
Draw reins (for lunging or riding)	○	○	○	○	○	○
Overcheck (checkrein, bearing rein)	○	○	○	○	○	○
A bosal (Western) or English hackamore	○	○	○	○	○	○
A crupper	○	○	○	○	○	○

Grass reins (anti-grazing reins)	Never	Rarely	Sometimes	Usually	Always	I don[illegible] kno[illegible] wha[illegible] tha[illegible] is

During the past 6 months, has ${e://Field/horsename} been afraid of the following?

	Never	Rarely	Sometimes	Usually	Always
Bicycles	○	○	○	○	○
Motor bikes, quad bikes, all-terrain vehicles or tractors	○	○	○	○	○
Cars, trucks or trailers	○	○	○	○	○
Horse drawn vehicles	○	○	○	○	○
Chain saws	○	○	○	○	○

	Never	Rarely	Sometimes	Usually	Always
Lawn mowers	○	○	○	○	○
Umbrellas	○	○	○	○	○
Tents	○	○	○	○	○
Plastic bags	○	○	○	○	○
Strollers/prams	○	○	○	○	○
Children	○	○	○	○	○
Dogs	○	○	○	○	○
Wild animals (deer, kangaroo)	○	○	○	○	○
Domestic animals (cow, sheep, chickens)	○	○	○	○	○
Uncommon domestic animals (alpacas, pigs)	○	○	○	○	○

Some horses display **defensive** or **aggressive** behavior in certain situations.

Typical **moderate signs** would include **threatening** to *bite*, *pinning ears*, *tail swishing*, threatening to *kick* or *strike*. The most **serious signs** would include **actual** *biting*, *kicking* or *striking*. Check a box on the 5-point scale below to indicate ${q://QID11/ChoiceTextEntryValue/4}'s recent tendency (using the past 6 months as a guide) to show these behaviors in the following context:

	1 - No signs	2	3	4	5 - Serious signs (kick, bite, strike)	Not observed/ applicable
When verbally corrected by you or another person on the ground	○	○	○	○	○	○
When verbally corrected when ridden/driven	○	○	○	○	○	○
When corrected with a leg or whip cue when ridden/driven	○	○	○	○	○	○
When approached by you in the paddock	○	○	○	○	○	○
When approached by you when tied/tethered	○	○	○	○	○	○

	1 - No signs	2	3	4	5 - Serious signs (kick, bite, strike)	Not observed/ applicable
When approached by you in the stable/stall or small enclosure	○	○	○	○	○	○
When approached by an unfamiliar person in the paddock	○	○	○	○	○	○
When approached by an unfamiliar person in the stable/stall or small enclosure	○	○	○	○	○	○
When approached by an unfamiliar person when tied/tethered	○	○	○	○	○	○
When approached by you when eating from a bucket or manger	○	○	○	○	○	○

	1 - No signs	2	3	4	5 - Serious signs (kick, bite, strike)	Not observed/ applicable
When approached by you carrying a bucket of feed	◯	◯	◯	◯	◯	◯
When a saddle is placed on his/her back	◯	◯	◯	◯	◯	◯
When the girth is done up	◯	◯	◯	◯	◯	◯
When hosed down	◯	◯	◯	◯	◯	◯
When approached by a familiar dog	◯	◯	◯	◯	◯	◯
When approached by a familiar animal of another species (goat, cat, rabbit, etc.)	◯	◯	◯	◯	◯	◯
When approached by an unfamiliar dog	◯	◯	◯	◯	◯	◯

	1 - No signs	2	3	4	5 - Serious signs (kick, bite, strike)	Not observed/ applicable
When approached by unfamiliar animal of another species (goat, cat, rabbit, etc.)	○	○	○	○	○	○
When being led towards an unfamiliar horse	○	○	○	○	○	○
When approached by an unfamiliar horse in the stall/stable or paddock/pasture	○	○	○	○	○	○
When being ridden/driven towards an unfamiliar horse	○	○	○	○	○	○
When being led beside an unfamiliar horse	○	○	○	○	○	○
When being ridden/driven beside or in front of an unfamiliar horse	○	○	○	○	○	○

	1 - No signs	2	3	4	5 - Serious signs (kick, bite, strike)	Not observed/ applicable
When being ridden/driven in a group of horses (i.e. trail ride, hacking)	○	○	○	○	○	○
When being ridden/driven in an arena with other horses (i.e. warm up, clinic, group lesson)	○	○	○	○	○	○
When being lunged or worked in a round pen	○	○	○	○	○	○
When signaled to go forward under saddle/in harness	○	○	○	○	○	○
When signaled to canter under saddle/in harness	○	○	○	○	○	○

	1 - No signs	2	3	4	5 - Serious signs (kick, bite, strike)	Not observed/ applicable
When signaled to make an upwards transition under saddle/in harness	○	○	○	○	○	○

Some horses show signs of anxiety when separated from other horses. Thinking back (over the past 6 months as a reference), how often does ${e://Field/horsename} show signs of each of the following behaviors when separated from other horses?

	Never	Rarely	Sometimes	Usually	Always	I don't know
Trembling	○	○	○	○	○	○
Sweating	○	○	○	○	○	○

T[illegible]

Trotting, cantering or galloping in the field	Never	Rarely	Sometimes	Usually	Always	I don't know
Jumping or pushing through a gate or fence						
Pacing, circling, rearing etc, in the stall						
Vocalizing (calling out or neighing)						
Pawing						
Pulling back when tied						

Does ${e://Field/horsename} show more anxiety when:

Some horses show signs of anxiety when away from home. Thinking back (over the past 6 months as a reference), how often does ${e://Field/horsename} show signs of each of the following behaviors when away from home (either when ridden or driven to a new location)?

	Never	Rarely	Sometimes	Usually	Always	Nc ob Ap
Restlessness	○	○	○	○	○	
Pacing	○	○	○	○	○	
Pulling at lead or reins	○	○	○	○	○	
Vocalizing	○	○	○	○	○	
Bucking	○	○	○	○	○	
Rearing	○	○	○	○	○	
Bolting	○	○	○	○	○	
Pulling back [illegible]	○	○	○	○	○	

when tied						
Moving about when tied	Never	Rarely	Sometimes	Usually	Always	Nc ob Ap
Other escape behaviors						

How often does ${e://Field/horsename}?

	Never	Rarely	Sometimes	Usually	Always	D kı
Wind-suck or crib-bite						
Weave						
Chew own rugs/blankets						
Chew other horses' rugs/blankets						
Chew wood (fence or stable)						

	Never	Rarely	Sometimes	Usually	Always	D k[illegible]
Chew bark						
Eat feces						
Eat soil						
Dunk hay in water						
Play with water						
Paw prior to feeding						
Paw when feeding						
Hold up one foot when feeding						
Kick out with a hind foot while eating						

	Never	Rarely	Sometimes	Usually	Always	D kr
Show signs of enjoying grooming (such as extending the neck or wiggling the upper lip)						
Unfasten gates						
Push gates open						
Box/stall walk						
Walk the fence line repeatedly						
Head shake (side to side)						
Head toss (up and down)						
Groan when being ridden						

	Never	Rarely	Sometimes	Usually	Always	D kı
Have its nose behind the vertical when ridden						
Yawn before, during or after work						
Respond with over-sensitivity to touch						
Lie down during work or handling						
Fall down in the stall						

In the past 6 months, apart from when you are there, how frequently has ${e://Field/horsename} been in the company of other horses (where they can touch and groom one another), whether in the paddock, field or housed indoors?

Thinking back over the past 6 months, how many times has ${e://Field/horsename}?

	Never	1 Time	2 Times	3 Times	4 Times	5 or Mor Tim[illegible]
Had a dental examination by a horse dentist or veterinarian	○	○	○	○	○	○
Been examined by a veterinarian for any reason (other than vaccinations or routine dental care)	○	○	○	○	○	○
Had body-work (chiropractic, massage etc.) done	○	○	○	○	○	○
Been shod	○	○	○	○	○	○
Had feet trimmed by a professional	○	○	○	○	○	○
Had feet trimmed by a non-professional	○	○	○	○	○	○
Been wormed/dewormed (with a commercial wormer such as a paste, drench, pellets)	○	○	○	○	○	○

Has ${e://Field/horsename} been vaccinated against Tetanus in the last 12 months?

Check 'yes' if boosters are up to date but 'no' if it's been 12 months or longer since last booster, even if it's booked in/scheduled.

Is ${e://Field/horsename} regularly vaccinated against any other diseases?

Such as Strangles, Hendra, West Nile etc.

Does ${e://Field/horsename}?

	Never	Rarely	Sometimes	Usually	Always
Come when called in the field	○	○	○	○	○

	Never	Rarely	Sometimes	Usually	Always
Avoid being caught (move away)	◯	◯	◯	◯	◯
Pull back when tied	◯	◯	◯	◯	◯
Throw head up when being bridled/haltered	◯	◯	◯	◯	◯
Step back when being bridled/haltered	◯	◯	◯	◯	◯
Throw head up when bridle/halter is removed	◯	◯	◯	◯	◯
Step back when bridled/halter is removed	◯	◯	◯	◯	◯
Load easily/without resistance onto the trailer	◯	◯	◯	◯	◯
Load easily/without resistance onto the truck	◯	◯	◯	◯	◯

	Never	Rarely	Sometimes	Usually	Always
Unload slowly off the trailer	◯	◯	◯	◯	◯
Rush off the trailer backwards when loading	◯	◯	◯	◯	◯
Rush off the trailer backwards when unloading	◯	◯	◯	◯	◯
Rush on to the trailer forwards when loading	◯	◯	◯	◯	◯
Unload slowly off the truck	◯	◯	◯	◯	◯
Fall when travelling in the trailer	◯	◯	◯	◯	◯
Kick when travelling in the trailer	◯	◯	◯	◯	◯
Vocalize when travelling in the trailer	◯	◯	◯	◯	◯

	Never	Rarely	Sometimes	Usually	Always
Walk into handler when led	◯	◯	◯	◯	◯
Push handler when offered food	◯	◯	◯	◯	◯

How often have you spent time with ${e://Field/horsename} over the past 6 months?

Include any activity 15 minutes or longer - grooming, feeding, visiting, petting, riding, exercising and ground work.

Powered by Qualtrics
